# Two *Enterococcus faecium* Isolates Demonstrated Modulating Effects on the Dysbiosis of Mice Gut Microbiota Induced by Antibiotic Treatment

**DOI:** 10.3390/ijms25105405

**Published:** 2024-05-15

**Authors:** Xiaohui Yao, Wansen Nie, Xi Chen, Junjie Zhang, Jianchao Wei, Yafeng Qiu, Ke Liu, Donghua Shao, Haixia Liu, Zhiyong Ma, Zongjie Li, Beibei Li

**Affiliations:** Shanghai Veterinary Research Institute, Chinese Academy of Agricultural Science, Shanghai 200241, China; 15160870804@163.com (X.Y.); niewansen@163.com (W.N.); chenxi992024@163.com (X.C.); 17317271403@163.com (J.Z.); jianchaowei@shvri.ac.cn (J.W.); yafengq@shvri.ac.cn (Y.Q.); liuke@shvri.ac.cn (K.L.); shaodonghua@shvri.ac.cn (D.S.); lhx@zgzkyk.com (H.L.); zhiyongma@shvri.ac.cn (Z.M.)

**Keywords:** antibiotic, probiotic, *Enterococcus faecium*, bacteriocin, short-chain fatty acids, gut microbiota

## Abstract

Broad-spectrum antibiotics are frequently used to treat bacteria-induced infections, but the overuse of antibiotics may induce the gut microbiota dysbiosis and disrupt gastrointestinal tract function. Probiotics can be applied to restore disturbed gut microbiota and repair abnormal intestinal metabolism. In the present study, two strains of *Enterococcus faecium* (named DC-K7 and DC-K9) were isolated and characterized from the fecal samples of infant dogs. The genomic features of *E. faecium* DC-K7 and DC-K9 were analyzed, the carbohydrate-active enzyme (CAZyme)-encoding genes were predicted, and their abilities to produce short-chain fatty acids (SCFAs) were investigated. The bacteriocin-encoding genes in the genome sequences of *E. faecium* DC-K7 and DC-K9 were analyzed, and the gene cluster of Enterolysin-A, which encoded a 401-amino-acid peptide, was predicted. Moreover, the modulating effects of *E. faecium* DC-K7 and DC-K9 on the gut microbiota dysbiosis induced by antibiotics were analyzed. The current results demonstrated that oral administrations of *E. faecium* DC-K7 and DC-K9 could enhance the relative abundances of beneficial microbes and decrease the relative abundances of harmful microbes. Therefore, the isolated *E. faecium* DC-K7 and DC-K9 were proven to be able to alter the gut microbiota dysbiosis induced by antibiotic treatment.

## 1. Introduction

The invention and widespread use of antibiotics have significantly contributed to the treatment of bacteria-induced infections. However, the side effects of antibiotic overuse should also not be neglected. In fact, frequent exposure to antibiotics in early life could disrupt the establishment and development of normal symbiotic microbiota [1,2]. Perturbations of commensal microbiota by antibiotic usage might lead to the colonization and expansion of pathogenic bacteria (such as *Clostridium difficile*, *C. perfringens*, *Klebsiella oxytoca*, *K. pneumonia*, and *Staphylococcus aureus*) and cause the occurrence of antibiotic-associated diarrhea (AAD) [3]. Moreover, extended antibiotic therapy could significantly decrease the structure of the infant gut microbiome and reduce the relative abundances of beneficial microbes (such as *Lactobacillus* and *Bifidobacterium*) [4,5,6]. Alterations of the gut microbiome caused by antibiotic administration might also increase the risks of asthma, allergies, obesity, type 1 diabetes, and inflammatory bowel disease (IBD) [1,7,8]. Therefore, the disruption of the gut microbiome by antibiotics in early life could increase the risks of metabolic and immunological diseases in later life.

When antibiotics are applied to inhibit colonic pathogens, the absorptions of luminal carbohydrates, short-chain fatty acids (SCFAs), NaCl, and water are also altered, and finally cause osmotically mediated diarrhea [9,10]. Antibiotic-induced gut microbiota dysbiosis could decrease the intestinal expressions of SCFA receptor (GPR109a), transporter (SLC5A8), and monocarboxylate transporter isoform 1 (MCT1), and inhibit the uptake of butyrate [11,12]. Moreover, antibiotics could also alter the intestinal pH and oxygen levels by reducing the relative abundances of gut microbes that could produce acetate, propionate, butyrate, and other organic acids [13]. Additionally, antibiotic-induced *C. difficile* infection (CDI) could disrupt the metabolism of luminal bile acids and enhance the colonic levels of primary and secondary bile acids that are associated with diarrhea [14,15]. Therefore, antibiotics could alter the intestinal metabolism of organic acids and bile acids by changing the gut microbial ecology and disrupt the transport process of water and solutes.

Many clinical trials have proven that co-administration of probiotics with antibiotics could restore the gut microbiota, repair the balance of the microbial communities in the gastrointestinal tract, and reduce the risk of *C. difficile* infection [16]. Single-strain or combination members of probiotics (such as *Bacillus*, *Bifidobacterium*, *Clostridium butyricum*, *Lactobacillus*, *Lactococcus*, *Leuconostoc cremoris*, *Saccharomyces*, and *Streptococcus*) were frequently used to prevent AAD, and the specified efficacy and underlying molecular mechanisms of probiotic action were also evaluated and investigated [17]. The immune homeostasis regulated by oral administration of *Lactobacillus* and other probiotics was associated with decreased systemic inflammatory responses (reduction in C-reactive protein, Complement C3, and IgG) and the activated immunomodulation function of immune cells [18,19]. Several probiotic strains (such as *B. amyloliquefaciens*, *L. casei* CGMCC 12435, and *Bacteroides fragilis* ZY-312) were proven to be able to improve intestinal barrier integrity and epithelial permeability, and the intestinal barrier function was associated with enhanced expressions of colonic tight junction proteins (such as ZO-1 and occludin) [8,20,21]. The bacteriocin Plantaricin EF, which is produced by *L. plantarum*, was found to be able to increase intestinal ZO-1 synthesis and protect intestinal barrier integrity [22]. A class of bacteriocins produced by *E. coli* Nissle 1917 were also proven to be able to prevent intestinal inflammation and inhibit the competitive exclusion of *Enterobacteriaceae* [23]. In addition, many strains of *Enterococcus* which inhabit human and animal guts are used as probiotics for humans, animals, and starter cultures in the food industry [24]. *E. faecalis* was proven to be an effective antibiotic alternative for the beneficial effects of enhancing animal health and growing performance [25]. Moreover, *E. faecium* were also found to be able to improve the host intestinal epithelial defense program and limit the pathogenesis of enteric infection induced by *Salmonella enterica* and *C. difficile* [26]. Kim et al. proved that the intestinal barrier function and pathogen tolerance which were improved by *E. faecium* were associated with the secretion of peptidoglycan hydrolase (SagA) [27]. Therefore, the single functional strain or a mixture of multiple probiotic strains were proven to be able to inhibit the systemic inflammatory response and improve the intestinal barrier function.

In the present study, two strains of *E. faecium* (named DC-K7 and DC-K9) were isolated and characterized, and their genomic features and SCFA-producing abilities were also investigated. Furthermore, the modulating effects of *E. faecium* DC-K7 and DC-K9 on the gut microbiota dysbiosis induced by antibiotics were also analyzed.

## 2. Results

### 2.1. Isolation and Characterization of E. faecium DC-K7 and DC-K9

**Figure 1:**
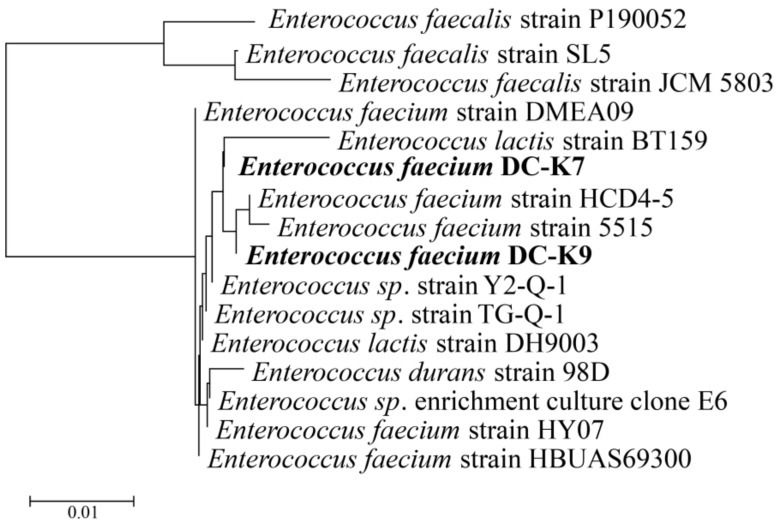
Phylogenetic tree of *E. faecium* DC-K7 and DC-K9 based on the neighbor-joining method of 16S rDNA gene sequences.

Two strains of lactic acid bacteria (named *E. faecium* DC-K7 and DC-K9) were isolated from infant dog guts using a selective de Man, Rogosa and Sharpe (MRS) broth. The sequences of 16S rDNA genes were amplified by PCR and compared by BLAST (www.ncbi.nlm.nih.gov/blast (accessed on 15 July 2023)) for genotypic analysis, using other known sequences deposited in the GenBank database. As shown in Figure 1, the neighbor-joining phylogenetic tree of different *Enterococcus* strains was generated using the Mega11 software (https://www.megasoftware.net/ (accessed on 15 July 2023)).

### 2.2. General Features of E. faecium DC-K7 and DC-K9 Genome

**Figure 2:**
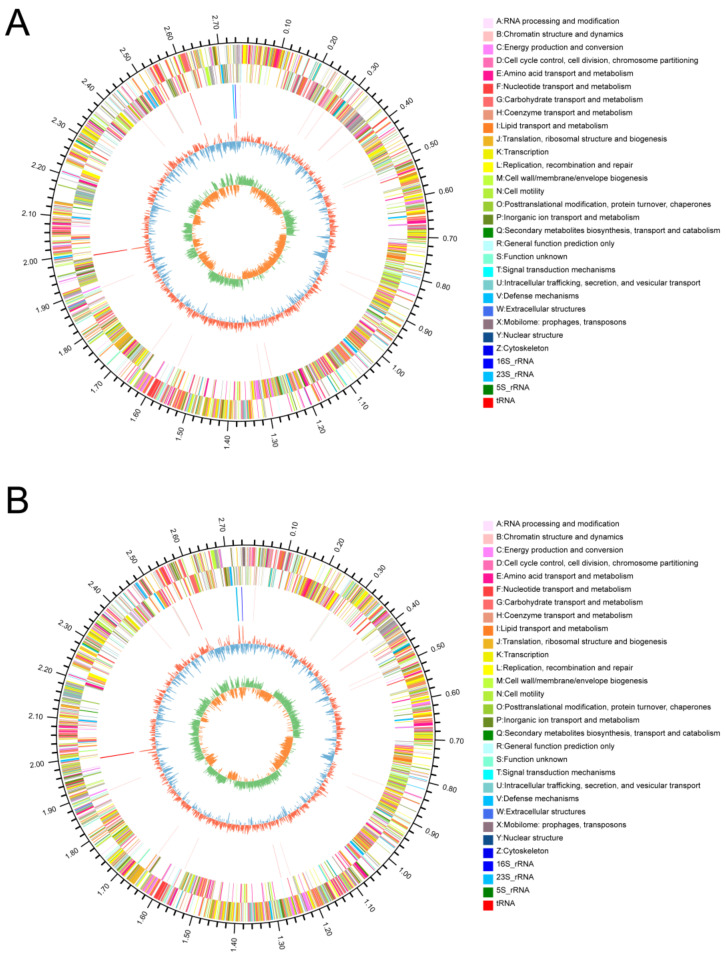
Circos maps of the *E. faecium* DC-K7 (**A**) and DC-K9 (**B**) genome. The outer circle represents the genome size in kb. The second and third circles represent the predicted coding sequences (CDSs), and the different colors indicate the different clusters of orthologous groups of proteins (COGs). The fourth circle represents the rRNA and tRNA clusters. The fifth circle represents the GC content with red and blue color, and the most inner circle represents GC-skew values.

A total of 8,230,232 and 5,980,392 high-quality reads were obtained from the genomes *E. faecium* DC-K7 and DC-K9, and their genome sequences were assembled into 62 contigs and 61 contigs, respectively (Table 1). The chromosome lengths of *E. faecium* DC-K7 and DC-K9 were 2,748,310 bp and 2,748,609 bp, with 452-fold and 328-fold average genome coverage and GC contents of 38.09%. Using the GeneMarks v4.32 software (http://topaz.gatech.edu/GeneMark/ (accessed on 15 July 2023)), 2707 and 2704 open reading frames (ORFs) were functionally annotated from the predicted protein coding sequences (Figure 2). The genome of *E. faecium* DC-K7 contained 5 rRNA operons and 57 tRNA genes, while the genome of *E. faecium* DC-K9 contained 6 rRNA operons and 57 tRNA genes (Table 1).

### 2.3. Analyses of CAZyme-Encoding Genes

The amounts of carbohydrate-active enzyme (CAZy)-encoding genes in the genome sequences of *E. faecium* DC-K7 and DC-K9 were almost the same. In all, 20 genes of Glycosyl Transferase (GT), 4 genes of Polysaccharide Lyase (PL), 16 genes of Carbohydrate Esterase (CE), 4 genes of Auxiliary Activities (AAs), 8 genes of Carbohydrate-Binding Modules (CBMs), and 56 genes of Glycoside Hydrolases (GHs) were predicted in the genome sequences of *E. faecium* DC-K7 and DC-K9 (Table 2).

### 2.4. Examinations of SCFA-Producing Abilities

The SCFAs produced by *E. faecium* DC-K7 and DC-K9 were quantified by Liquid Chromatography–Mass Spectrometry (LC-MS) (Table 3). The concentrations of SCFAs produced by *E. faecium* DC-K7 and DC-K9 were measured as follows: acetic acid (4327.02 vs. 2329.69 µM), propanoic acid (37.12 vs. 30.70 µM), isobutyric acid (13.41 vs. 11.93 µM), butyric acid (2.96 vs. 2.47 µM), isovaleric acid (2.04 vs. 1.24 µM), valeric acid (1.10 vs. 1.73 µM), 4-methylvaleric acid (0.13 vs. 0.08 µM), and caproic acid (6.16 vs. 5.38 µM).

### 2.5. Bacteriocin Prediction

**Figure 3:**
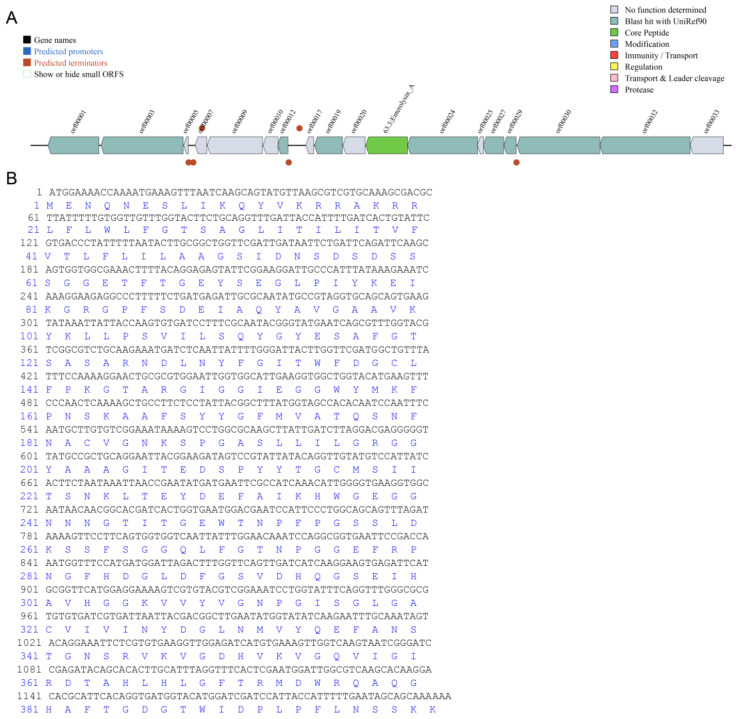
Bacteriocin predicted by the BAGEL4 software. Genes are indicated as arrows and the expression data are displayed in RPKM (**A**), with the nucleotide sequence of Enterolysin-A and the deduced amino acid sequence (**B**).

Using the software of BAGEL4 (http://bagel4.molgenrug.nl (accessed on 15 July 2023)), the bacteriocin-encoding genes in the genome sequences of *E. faecium* DC-K7 and DC-K9 were predicted. As shown in Figure 3A, the identified gene cluster is visualized in reads per kilobase million (RPKM). A core peptide named Enterolysin-A was predicted, and the peptide was composed of 401 amino acid residues with a calculated molecular weight of 43.18 kDa. The nucleotide sequence and the deduced amino acid sequence are shown in Figure 3B.

### 2.6. Microbial Diversity Analysis of the Mice Gut Microbiota

**Figure 4:**
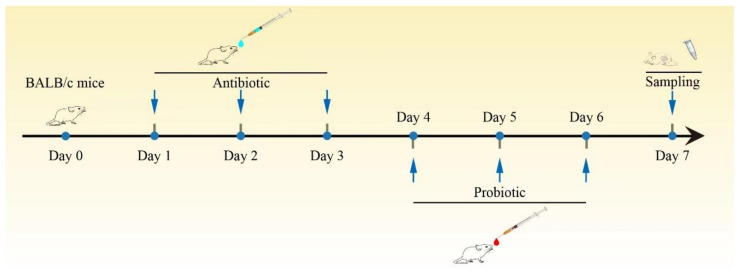
Experimental outline. The BALB/c mice were treated with amoxicillin by oral gavage at a dose of 50 mg/kg for 3 days, and then were given the cultured probiotics (2 × 10^8^ CFU/mL) by oral gavage for another 3 days. At the end of the experiment trial, all the mice fecal samples were collected.

In the present study, the gut dysbiosis mouse model was established by oral administration of amoxicillin (at a dose of 50 mg/kg) and was then treated by oral administration of probiotics (2 × 10^8^ CFU/mL). The fecal samples of mice in the three groups were collected and the gut microbiota was evaluated by 16S rDNA sequencing (Figure 4).

**Figure 5:**
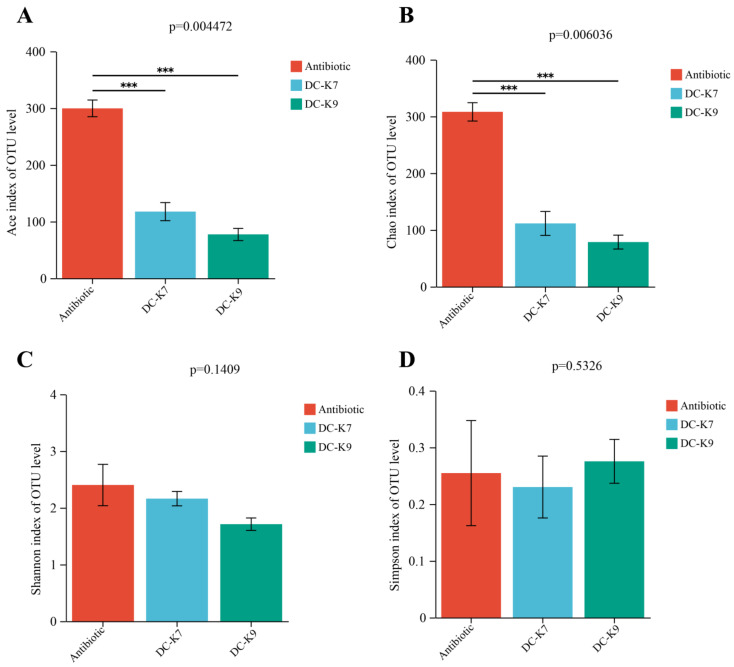
The alpha diversity of gut microbiota in mice. The richness of the mice gut microbiota was indicated by the ACE estimators (**A**) and the Chao estimators (**B**), and the diversity of the mice gut microbiota was indicated by the Shannon estimators (**C**) and the Simpson estimators (**D**). *** *p* < 0.01.

The alpha diversity of gut microbiota was analyzed using the Abundance-based Coverage Estimator (ACE) estimators, the Chao estimators, the Shannon estimators, and the Simpson estimators, respectively (Figure 5).

When compared to the antibiotic group, the ACE estimators and the Chao estimators revealed that the abundances of gut microbiota in the *E. faecium* DC-K7 and DC-K9 group were obviously decreased (Figure 5A,B). The Shannon estimators and the Simpson estimators demonstrated that the diversities of gut microbiota in the *E. faecium* DC-K7 and DC-K9 group were lower than the antibiotic group; however, the differences were not significant (Figure 5C,D). Therefore, the alpha diversity analysis revealed that the richness and diversity of the mice gut microbiota were both decreased by the *E. faecium* DC-K7 and DC-K9 treatments.

**Figure 6:**
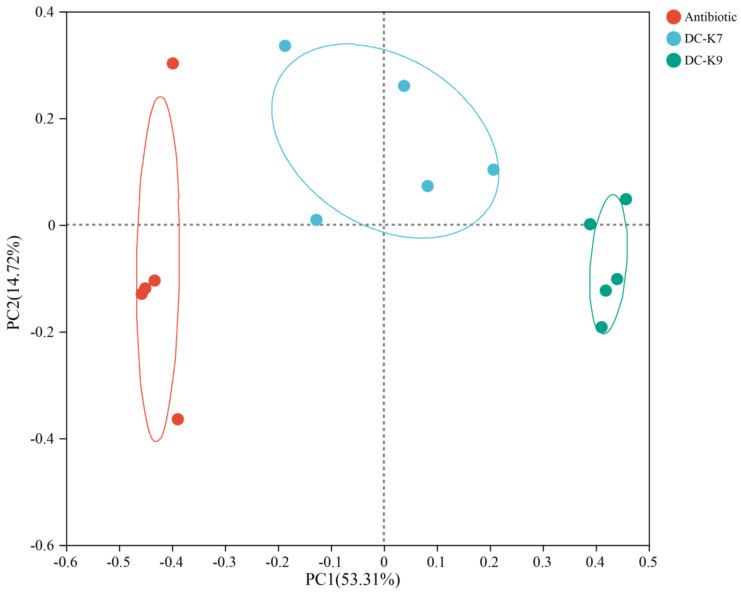
The beta diversity analysis of the fecal microbiota. A plot of the principal coordinate analysis was created, and the results demonstrated that the microbial communities of the three groups were separated into different clusters.

For the beta diversity analysis of the gut microbiota in mice, a principal coordinate analysis (PCoA) was performed. As shown in Figure 6, the microbial communities of the three groups were segregated into different clusters, which revealed that the beta diversities of the three groups were quite different.

### 2.7. Alterations of the Gut Microbial Compositions

**Figure 7:**
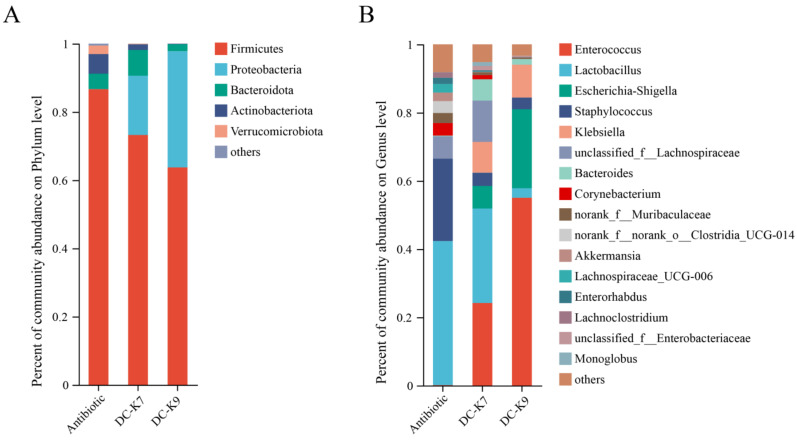
The bacterial communities at the phylum (**A**) and genus (**B**) levels. Relative abundances of the phyla or genera less than 1% were merged into others.

With the aim to assign the bacterial taxonomic communities of the gut microbiota in mice, the RDP classifier was used to compare the gut microbial compositions of the three groups at the phylum level and the genus level, respectively (Figure 7).

As shown in Table 4, the predominant microbial communities of the three groups at the phylum level were Firmicutes (86.68% vs. 73.22% vs. 63.72%), Proteobacteria (0.03% vs. 17.37% vs. 34.05%), Bacteroidota (4.51% vs. 7.56% vs. 2.06%), Actinobacteriota (5.75% vs. 1.66% vs. 0.13%), and Verrucomicrobiota (2.50% vs. 0.14% vs. 0.01%), respectively.

As shown in Table 5, the predominant microbial communities of the three groups at the genus level were Enterococcus (0.12% vs. 24.20% vs. 55.07%), Lactobacillus (42.23% vs. 27.69% vs. 2.77%), Escherichia-Shigella (0.01% vs. 6.63% vs. 23.17%), Staphylococcus (24.13% vs. 3.87% vs. 3.42%), Klebsiella (0.00% vs. 9.05% vs. 9.64%), Bacteroides (0.20% vs. 6.24% vs. 1.65%), Corynebacterium (3.69% vs. 1.23% vs. 0.02%), Akkermansia (2.50% vs. 0.14% vs. 0.01%), Lachnospiraceae_UCG-006 (2.54% vs. 0.08 vs. 0.00%), Enterorhabdus (1.78% vs. 0.34% vs.0.07%), Lachnoclostridium (1.61% vs. 0.08% vs. 0.05%), and Monoglobus (8.11% vs. 5.20% vs. 3.33%), respectively.

**Figure 8:**
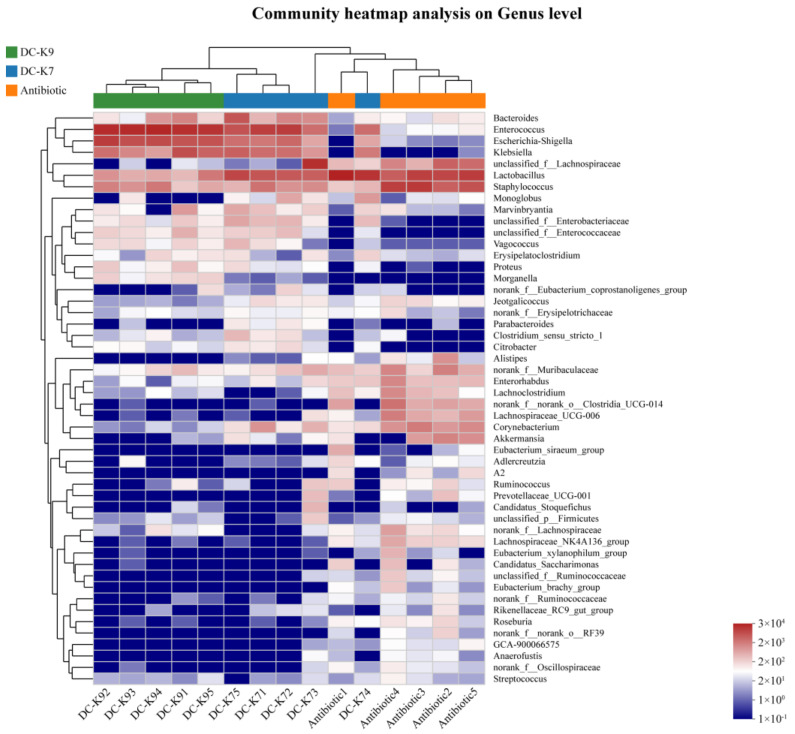
Heatmap analysis of the gut microbiota.

Moreover, a heatmap was generated to demonstrate the hierarchy cluster results for the abundances of genera, and the top 50 genera of the microbial communities in each sample are shown (Figure 8). The color of the spots corresponds to the normalized and log-transformed relative abundances, and the genus names are shown on the right.

### 2.8. Comparisons of the Gut Microbial Communities

**Figure 9:**
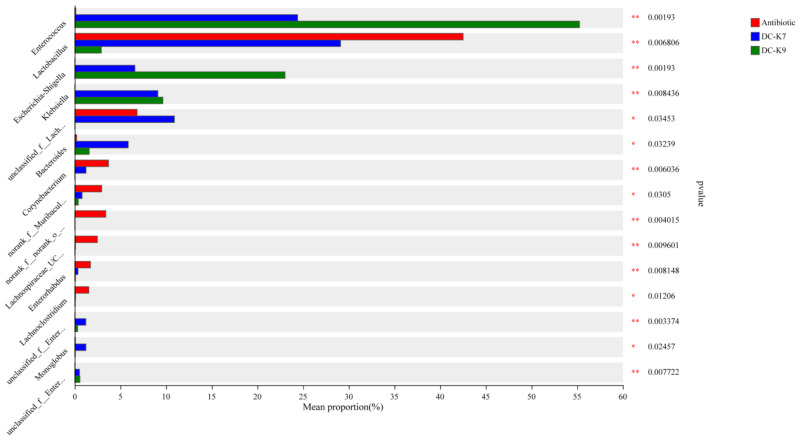
The different bacterial taxa among the three groups were compared. The ordinate indicates the bacterial name, and the abscissa indicates the percentage value. * *p* < 0.05 and ** *p* < 0.01 were considered statistically significant.

The significant differences in the microbial communities were analyzed by the Kruskal–Wallis test, and the relative abundances of the predominant genera among the three groups were calculated and compared. As shown in Figure 9, the relative abundances of Enterococcus and Bacteroides in the *E. faecium* DC-K7 group and *E. faecium* DC-K9 group were significantly higher than the antibiotic group. However, the relative abundances of Corynebacterium and Enterorhabdus in the *E. faecium* DC-K7 group and *E. faecium* DC-K9 group were significantly lower than those in the antibiotic group. The obvious alterations of gut microbial communities might be related to the regulating effects of *E. faecium* DC-K7 and DC-K9 on the gut microbiota dysbiosis induced by antibiotics.

## 3. Discussion

Lactic acid bacteria (LAB) are widely distributed in the animal gut, fermented foods, and the natural environment. LAB are commonly considered as potential probiotics based on their ability to produce lactic acid and other beneficial metabolites [28].

Previous studies have proven that *Enterococcus* could protect against the gut microbiota dysbiosis induced by antibiotics, and the improved gastrointestinal tract function and the altered gut microbiota could decrease the peripheral inflammation and stress response [29,30]. In fact, *Enterococcus* strains commonly exist in traditional functional foods and play an important role in maintaining the characteristic taste and flavor of Mediterranean food [24]. Moreover, the commensal *Enterococcus* strains that inhabit the human and animal gastrointestinal tract could regulate the host’s energy metabolism and protect the gut barrier function [31]. In the current study, two strains of *E. faecium* (named DC-K7 and DC-K9) were isolated and characterized, and their modulating effects on gut microbiota dysbiosis were analyzed.

In fact, the richness and diversity of gut microbial communities change rapidly in different life periods [32]. In early life, the formation and maturation of the intestinal microbiota can be influenced by many factors, and the beneficial microbes in the infant gut are much higher than those in the adult gut [33]. Therefore, it is much more possible to isolate and obtain probiotics from infant gut samples. Fecal samples of mammals are frequently used to indicate gut microbiota because the bacterial profiles of paired faecal and rectal biopsy wash samples are very similar [34]. In the present study, two strains of *E. faecium* (named *E. faecium* DC-K7 and K9) were isolated from the gut microbiota of infant dogs, and their 16S rDNA genes were amplified by PCR and compared by BLAST. As shown in Figure 1, the 16S rDNA nucleotide sequences of two *E. faecium* strains displayed a very high similarity to other *Enterococcus* strains deposited in NCBI. The phylogenetic analysis demonstrated that both strains were closely related to the *E. faecium* strain HCD4-5 and *E. faecium* strain 5515. The general genomic features of *E. faecium* DC-K7 and DC-K9 are shown in Table 1, and their genome maps are shown in Figure 2. Studies on the genomic characteristics of the two *E. faecium* strains provide novel insights into their functional genes.

The CAZy-encoding genes in the genome sequences of *E. faecium* DC-K7 and DC-K9 were analyzed. Interestingly, the numbers of CAZy-encoding genes in the genome sequences of the two strains of *E. faecium* were the same. The CAZymes were associated with carbohydrate fermentation and degradation, and the produced lactic acid and SCFAs could regulate the pH of the gastrointestinal tract and inhibit the colonization of opportunistic pathogens [35]. The SCFA-producing properties of *E. faecium* DC-K7 and DC-K9 were examined using GC/TOF MS, and the concentrations of acetic acid, propanoic acid, isobutyric acid, butyric acid, isovaleric acid, valeric acid, 4-methylvaleric acid, and caproic acid were measured, respectively. The contents of acetic acid in the culture medium of *E. faecium* DC-K7 and DC-K9 were much higher than other SCFAs (Table 3). A previous study proved that Bifidobacterium, Enterococcus, and other probiotics could enhance the abundance of SCFA-producing bacteria in the gut microbiota of patients with diarrhea-predominant irritable bowel syndrome (IBS-D), and their fecal concentrations of SCFAs were also increased by probiotic treatment [36]. Moreover, the abdominal pain response and stool consistency of patients with IBS-D could be improved by oral administration of *E. faecalis* (DSM 16440) and *E. coli* (DSM 17252) [37].

In fact, *Enterococcus* treatments could ameliorate the gut barrier function and protect the immune system by inhibiting invading pathogens (such as Staphylococcus aureus and others) [31,38]. The alterations of the gut microbiota by *Enterococcus* treatment might be associated with the produced bacteriocins and other secondary metabolites [39,40]. In the genome sequences of *E. faecium* DC-K7 and DC-K9, the gene clusters which encoded Enterolysin A were identified (Figure 3). For the reason that broad-spectrum antibiotic treatment could change the gut microbiota composition and suppress cellular and functional systemic immune development, probiotics were frequently used to restore the gut microbiota and regulate immune homeostasis [5,6,7,41]. In the current study, the modulating effects of *E. faecium* DC-K7 and DC-K9 on gut microbiota dysbiosis were investigated. When compared to the antibiotic group, the abundances of gut microbiota in the *E. faecium* DC-K7 and DC-K9 groups were obviously decreased (Figure 5A,B); however, the diversities of gut microbiota in the *E. faecium* DC-K7 and DC-K9 groups were not changed significantly (Figure 5C,D). For beta diversity analysis, the microbial communities of the three groups were obviously segregated into different clusters, which revealed that the beta diversities of the gut microbiota were significantly changed by the *E. faecium* DC-K7 and DC-K9 treatments (shown in Figure 6). Moreover, the gut microbial compositions of the *E. faecium* DC-K7 and DC-K9 groups were altered at the phylum and genus level when compared to the antibiotic group (Figure 7). At the genus level, the relative percentages of Enterococcus in the *E. faecium* DC-K7 and DC-K9 groups were obviously enhanced when compared to the antibiotic group (Table 5), which revealed that *Enterococcus* colonized well in the intestinal tract. In the current study, the colonization of *Enterococcus* influenced the relative percentages of *Lactobacillus* in the mice gut microbiota. In fact, *Enterococcus* and *Lactobacillus* are two predominant lactic acid bacteria in the gastrointestinal tract, and they have certain competitive relationships in the acquisition of the ecological site and nutritional supply [42]. Meanwhile, the relative percentages of Bacteroides and Escherichia-Shigella were also obviously enhanced in the *E. faecium* DC-K7 and DC-K9 groups. However, the relative percentages of *Staphylococcus*, *Corynebacterium*, and *Enterorhabdus* were obviously decreased when compared to the antibiotic group (Table 5). Consistent with previous studies [43,44], the current study shows that oral administrations of *E. faecium* DC-K7 and DC-K9 could alter the gut microbiota by enhancing the relative percentages of beneficial microbes and decreasing the relative percentages of harmful microbes. Therefore, the current study demonstrated that the gut microbiota dysbiosis induced by antibiotics was repaired by the *E. faecium* DC-K7 and DC-K9 treatments (Figure 8 and Figure 9).

## 4. Materials and Methods

### 4.1. Isolation and Characterization of E. faecium DC-K7 and DC-K9

The LAB strains were originally isolated from the fresh feces of infant dogs which were recruited at the Shanghai Longgen Working Dog Center, and then the isolated LAB strains were cultured in MRS broth for 48 h at 37 °C under anaerobic conditions [45]. The isolated *E. faecium* DC-K7 and DC-K9 strains were further characterized by 16S rDNA gene sequencing with primers 27F (5′-AGAGTTTGATCCTGGCTCAG-3′) and 1492R (5′-CTACGGCTACCTTGTTACGA-3′), and the species were initially determined by the BLAST program in the National Center for Biotechnology Information (NCBI) (http://www.ncbi.nlm.nih.gov/ (accessed on 15 July 2023)). The phylogenetic tree was generated using the MEGA11 package [46].

### 4.2. Genomic Features of E. faecium DC-K7 and DC-K9

#### 4.2.1. Genome Sequencing, Assembly, CDS Prediction and Annotation

DNA was extracted using a bacterial DNA extraction kit (TIANGEN Biotech CO., Ltd., Beijing, China) according to the manufacturer’s instructions. Whole Genome Shotgun (WGS) sequencing was carried out using an Illumina NovaSeq sequencing platform (Illumina Inc., San Diego, CA, USA) by constructing 2 × 150 bp paired-end libraries and generating 400 bp reads. The obtained paired-end reads were then assembled de novo into contigs and scaffolds using A5-MiSeq v20160825 (https://arxiv.org/abs/1401.5130 (accessed on 15 July 2023)) and SPAdes v3.12.0 (http://cab.spbu.ru/files/release3.12.0/manual.html (accessed on 15 July 2023)) [47,48]. The coding sequences (CDSs) were predicted using GeneMarkS and the functional annotation of the sequenced genome was achieved using Diamond v2.0.11 (http://github.com/bbuchfink/diamond (accessed on 15 July 2023)) against the NCBI database [49].

#### 4.2.2. Prediction of CAZyme-Encoding Genes

The potential CAZyme-encoding genes in the genomes of *E. faecium* DC-K7 and DC-K9 were predicted using the HMMSCAN software package (http://hmmer.org/ (accessed on 10 April 2023)) [35]. The CAZyme database (http://www.cazy.org (accessed on 10 April 2023)) was used to query the ORFs of Glycoside Hydrolases (GHs), Glycosyl Transferases (GTs), Polysaccharide Lyases (PLs), Carbohydrate Esterases (CEs), Auxiliary Activities (AAs), and Carbohydrate-Binding Modules (CBMs).

#### 4.2.3. Prediction of Bacteriocin

A user-friendly web server (BAGEL4: http://bagel4.molgenrug.nl (accessed on 27 August 2023)) was used to predict the ribosomally synthesized and post-translationally modified peptides (RiPPs) and bacteriocins in the genome sequences of *E. faecium* DC-K7 and DC-K9 [35,50]. The identified gene clusters of interest were discovered using the core-peptide database, and the deduced amino acid sequence was translated using online biological software (http://www.bio-soft.net/sms/index.html (accessed on 27 August 2023)).

### 4.3. Examination of SCFA-Producing Abilities

The *E. faecium* DC-K7 and DC-K9 strains were cultured in MRS medium for 24 h and were centrifuged for 10 min at 6000 rpm. Then, supernatants were collected and the concentrations of SCFAs were determined by LC-MS analysis [51].

### 4.4. Animal Studies

A total of 24 BALB/c mice (8-week-old, male) were purchased from the Chinese Academy of Science (Shanghai, China); all the mice were housed in a room maintained at a temperature of 22 ± 1 °C and a humidity of 60 ± 5%, with air exchanged 12 times per h and a 12 h light/dark cycle. The mice were randomly divided into three groups (n = 8): antibiotic group, DC-K7 group, and DC-K9 group. The antibiotic-induced dysbiosis mouse model was given amoxicillin at a dose of 50 mg/kg by oral gavage for 3 days. The mice in the antibiotic group were given 200 µL of MRS medium by oral gavage for another 3 days, while the mice in the DC-K7 group and DC-K9 group were given 200 µL of cultured *E. faecium* DC-K7 and DC-K9 (2 × 10^8^ CFU/mL) by oral gavage for another 3 days, respectively. On the morning of day 7, the mice fecal samples of the three groups were collected and stored in sterile cryo-tubes at −80 °C.

### 4.5. Microbial Community Profiling

The bacterial genomic DNA was extracted using the E.Z.N.A.^®^ soil DNA Kit (Omega Bio-tek, Norcross, GA, USA) [52]. The quality of the extracted DNA was verified by agarose gel electrophoresis, and the DNA concentration was measured using spectrophotometry (NanoDrop, Thermo Scientific, Tokyo, Japan). The V3/V4 hypervariable regions of the bacterial 16S rDNA gene were amplificated using the following primers: 338F (5′-ACTCCTACGGGAGGCAGCAG-3′) and 806R (5′-GGACTACHVGGGTWTCTAAT-3′). The amplicons were used to construct DNA libraries and were sequenced using an Illumina NovaSeq PE250 platform (Illumina, San Diego, CA, USA) which was performed by Majorbio Bio-Pharm Technology Co., Ltd. (Shanghai, China) [53]. The sequenced raw reads were demultiplexed and quality-filtered by fastp version 0.20.0 (https://github.com/OpenGene/fastp accessed on 15 July 2023), and the high-quality reads were clustered into operational taxonomic units (OTUs) with 97% sequence similarity. The bioinformatic analysis was performed using the Quantitative Insights into Microbial Ecology (QIIME) package version 2 (http://qiime.org/install/index.html, accessed on 15 July 2023) [54].

### 4.6. Statistical Analysis

All results are presented as mean  ±  SD of triplicate measurements. Differences were analyzed by one-way ANOVA, with *p*-values  <  0.05 considered statistically significant. In the comparison of the microbial diversity index and relative abundance, the Kruskal–Wallis test or Wilcoxon rank sum test were used, and *p* < 0.05 was determined statistically significant for statistical analyses.

## 5. Conclusions

In conclusion, the isolated *E. faecium* DC-K7 and DC-K9 strains demonstrated potential probiotic efficacies to modulate the gut microbiota dysbiosis induced by antibiotic treatments. By producing short-chain fatty acids, bacteriocin, and other antibacterial metabolites, the *E. faecium* DC-K7 and DC-K9 strains could inhibit the colonization of opportunistic pathogens and restore the disrupted gut microbiota.

## Figures and Tables

**Table 1:** General genome features of *E. faecium* DC-K7 and DC-K9.

Strain		DC-K7	DC-K9
Size (bp)		2,748,310	2,748,609
GC content (%)		38.09	38.09
ORFs	Protein-coding genes (CDSs)	2707	2704
	Gene density (gene per kb)	0.985	0.984
	Average gene length (bases per gene)	869	870
	ORF/Genome (%)	85.63	85.60
RNAs	rRNAs (16S-23S-5S)	5	6
	tRNAs	57	57

**Table 2:** Analyses of CAZyme-encoding genes.

CAZymes	DC-K7	DC-K9
Glycosyl Transferase (GT)	20	20
Polysaccharide Lyase (PL)	4	4
Carbohydrate Esterase (CE)	16	16
Auxiliary Activities (AAs)	4	4
Carbohydrate-Binding Module (CBM)	8	8
Glycoside Hydrolase (GH)	56	56

**Table 3:** Measurements of SCFAs.

SCFAs	DC-K7 (µM)	DC-K9 (µM)
Acetic acid	4327.02 ± 287.02	2329.69 ± 161.42
Propionic acid	37.12 ± 3.57	30.70 ± 2.69
Isobutyric acid	13.41 ± 2.07	11.93 ± 2.93
Butyric acid	2.96 ± 0.24	2.47 ± 0.28
Isovaleric acid	2.04 ± 0.12	1.24 ± 0.14
Valeric acid	1.10 ± 0.45	1.73 ± 0.25
4-Methylvaleric acid	0.13 ± 0.03	0.08 ± 0.04
Caproic acid	6.16 ± 1.45	5.38 ± 1.04

**Table 4:** The bacterial compositions of gut microbiota at the phylum level.

Phyla	Antibiotics	DC-K7	DC-K9
Firmicutes	86.68%	73.22%	63.72%
Proteobacteria	0.03%	17.37%	34.05%
Bacteroidota	4.51%	7.56%	2.06%
Actinobacteriota	5.75%	1.66%	0.13%
Verrucomicrobiota	2.50%	0.14%	0.01%
others	0.54%	0.04%	0.02%

**Table 5:** The bacterial compositions of gut microbiota at the genus level.

Genera	Antibiotics	DC-K7	DC-K9
*Enterococcus*	0.12%	24.20%	55.07%
*Lactobacillus*	42.23%	27.69%	2.77%
*Escherichia-Shigella*	0.01%	6.63%	23.17%
*Staphylococcus*	24.13%	3.87%	3.42%
*Klebsiella*	0.00%	9.05%	9.64%
*unclassified_f__Lachnospiraceae*	6.58%	12.11%	0.03%
*Bacteroides*	0.20%	6.24%	1.65%
*Corynebacterium*	3.69%	1.23%	0.02%
*norank_f__Muribaculaceae*	2.92%	0.82%	0.40%
*norank_f__norank_o__Clostridia_UCG-014*	3.50%	0.00%	0.00%
*Akkermansia*	2.50%	0.14%	0.01%
*Lachnospiraceae_UCG-006*	2.54%	0.08%	0.00%
*Enterorhabdus*	1.78%	0.34%	0.07%
*Lachnoclostridium*	1.61%	0.08%	0.05%
*unclassified_f__Enterobacteriaceae*	0.00%	1.20%	0.32%
*Monoglobus*	0.07%	1.12%	0.06%
others	8.11%	5.20%	3.33%

## Data Availability

The complete genome sequences of *E. faecium* DC-K7 and K9 have been deposited in a database under the accession numbers of SRR27883400 and SRR27883856, and the raw reads of high-throughput sequencing data were deposited into the NCBI Sequence Read Archive (SRA) database (Accession Number: PRJNA992919).
